# New Phosphospecific Antibody Reveals Isoform-Specific Phosphorylation of CPEB3 Protein

**DOI:** 10.1371/journal.pone.0150000

**Published:** 2016-02-25

**Authors:** Lech Kaczmarczyk, Étienne Labrie-Dion, Kapil Sehgal, Marc Sylvester, Magdalena Skubal, Michele Josten, Christian Steinhäuser, Paul De Koninck, Martin Theis

**Affiliations:** 1 Institute of Cellular Neurosciences, Medical Faculty, University of Bonn, Bonn, Germany; 2 Département de Biochimie, de Microbiologie et de Bio-informatique, Université Laval, Québec, QC, Canada; 3 Institut universitaire en santé mentale de Québec, Québec, QC, Canada; 4 Institute of Biochemistry and Molecular Biology, University of Bonn, Bonn, Germany; 5 Institute of Medical Microbiology, Immunology and Parasitology, University of Bonn, Bonn, Germany; CNRS UMR7275, FRANCE

## Abstract

Cytoplasmic Polyadenylation Element Binding proteins (CPEBs) are a family of polyadenylation factors interacting with 3’UTRs of mRNA and thereby regulating gene expression. Various functions of CPEBs in development, synaptic plasticity, and cellular senescence have been reported. Four CPEB family members of partially overlapping functions have been described to date, each containing a distinct alternatively spliced region. This region is highly conserved between CPEBs-2-4 and contains a putative phosphorylation consensus, overlapping with the exon seven of CPEB3. We previously found CPEBs-2-4 splice isoforms containing exon seven to be predominantly present in neurons, and the isoform expression pattern to be cell type-specific. Here, focusing on the alternatively spliced region of CPEB3, we determined that putative neuronal isoforms of CPEB3 are phosphorylated. Using a new phosphospecific antibody directed to the phosphorylation consensus we found Protein Kinase A and Calcium/Calmodulin-dependent Protein Kinase II to robustly phosphorylate CPEB3 *in vitro* and in primary hippocampal neurons. Interestingly, *status epilepticus* induced by systemic kainate injection in mice led to specific upregulation of the CPEB3 isoforms containing exon seven. Extensive analysis of CPEB3 phosphorylation *in vitro* revealed two other phosphorylation sites. In addition, we found plethora of potential kinases that might be targeting the alternatively spliced kinase consensus site of CPEB3. As this site is highly conserved between the CPEB family members, we suggest the existence of a splicing-based regulatory mechanism of CPEB function, and describe a robust phosphospecific antibody to study it in future.

## Introduction

**Fig 1:**
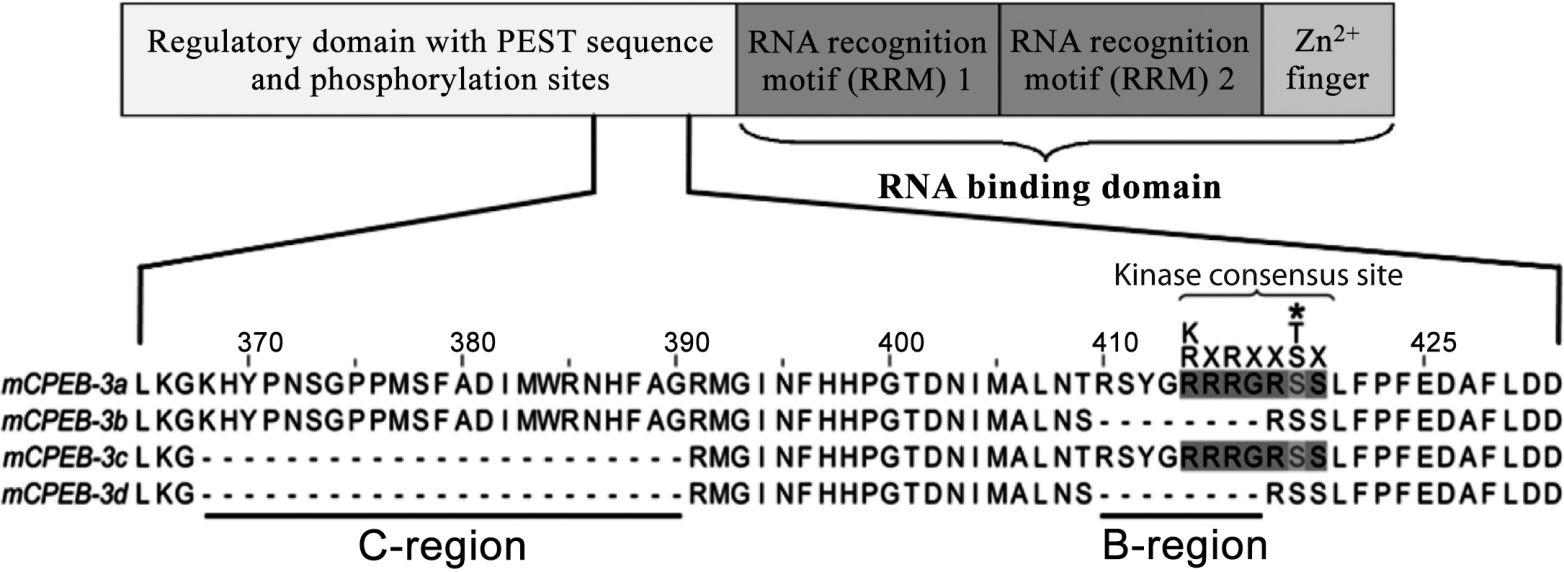
Schematic diagram of CPEB domain structure and sequence comparison of the variable middle region of mouse CPEB3. Two regions, termed B and C, are alternatively spliced, leading to expression of four isoforms: a (containing both regions), b (lacking the B-region), c (lacking the C-region), and d (lacking both regions) [Uniprot Q7TN99-1, -2, -3, -4 respectively]. Consensus phosphorylation sites for PKA and CaMKII was shown above the alignment. The putative phosphorylated residue was marked with an asterisk.

CPEBs are a family of RNA-binding proteins regulating translation [1]. They bind U-rich sequence motifs called Cytoplasmic Polyadenylation Elements (CPEs) in the 3’UTR of target transcripts [2, 3] (Fig 1). CPEBs were first described in *Xenopus laeavis* as translational activators of otherwise dormant mRNA during oocyte maturation [4, 5]. However, the role of CPEBs is not restricted to germ cells and 20% of the mammalian genome contains putative CPE elements [6]. Likewise, CPEB functions have expanded to mammalian development [7–9], cellular senescence [10–13], synaptic plasticity [14–18], cell migration [19] and others.

Four distinct CPEB paralogs have been described in mammals, each comprising a C-terminal RNA binding domain and an N-terminal regulatory domain with phosphorylation sites (Fig 1). CPEBs-2-4 represent a distinct subfamily, having >98% similarity in the RNA binding domain (and thereby target specificity), similar splicing patterns [20, 21], and common miRNA regulatory motifs [22]. Interestingly, CPEBs-2-4 have overlapping target mRNA specificity not only between themselves, but also with CPEB-1 [10, 21, 23–26].

In contrast to *Xenopus* CPEB, and its mouse homolog CPEB-1, much less information is available on how CPEBs-2-4 are regulated at the molecular level. Many mechanisms have been proposed, including phosphorylation [27], ubiquitination [16, 28], SUMOylation [29], modulation of subcellular localization [30] and micro RNAs [22]. All four CPEB paralogs are subject to alternative splicing [20, 21, 25, 31], but the biological significance of this phenomenon has never been addressed. Splicing of CPEB3 at exons 5 (partial skipping) and 7 (skipping) gives rise to 4 splice isoforms: a (full length—716aa, Uniprot Q7TN99-1), b (lacking 69nt/23aa fragment referred herein as the B-region, Uniprot Q7TN99-2), c (lacking 24nt/8aa fragment referred herein as the C-region, Uniprot Q7TN99-3), d (lacking both before B- and C-region, Uniprot Q7TN99-4) [20, 21] (Fig 1). This splicing pattern is conserved between CPEBs-2-4, although the size of the C-region differs [20, 21]. The remaining splice variants of CPEB3 are a 216aa N-terminal truncation (due to intra-exon skipping of exon 4) and a 123 aa C-terminal truncation (due to extension of exon 11 and earlier termination codon) [20]. We previously suggested a potential alternative splicing-based regulation of phosphorylation propensity for the two serine residues adjacent to the B-region of CPEBs-2-4 (Fig 1). This region contains a part of a putative phosphorylation consensus site for Protein Kinase A (PKA), Protein Kinase B (PKB), Calcium/Calmodulin-dependent Protein Kinase II (CaMKII) and Ribosomal Protein S6 Kinase (RPS6K), as determined *in silico* [20, 21].

Herein, we show that the serines S419 and S420 located downstream of the B-region (see Fig 1) are phosphorylated. *In vitro*, we observed a robust phosphorylation of the CPEB3-derived peptides, which was not detected when the B-region was absent. Using a phosphospecific antibody directed to the phosphorylation consensus, we tested the phosphorylation in several stimulation paradigms, using in cultured HEK-293 cells and primary hippocampal neurons. Finally, with special focus on the B-region, we performed *in vitro* screening of CPEB3a against 190 serine/threonine kinases, revealing putative candidate kinases targeting CPEB3a. In addition, by assessing phosphorylation of full-length CPEB3a using nanoflow liquid chromatography–mass spectrometry (LC-MS) we identified additional sites targeted by endogenous kinases in HEK-293 cells. The alternatively spliced region and the overlapping phosphorylation site are highly conserved between CPEBs-2-4, and we suggest that this site is implicated in regulatory functions of these proteins.

## Materials and Methods

### Animal handling

Maintenance and handling of animals used in this study was according to local government regulations and the European Communities Directive of 24 November 1986 (86/609/EEC). Experiments have been approved by the State Office of North Rhine-Westphalia, Department of Nature, Environment and Consumerism (LANUV NRW), approval number 84–02.04.2015.A393. All measures were taken to minimize the number of animals used, as well as to maximally reduce their discomfort and pain.

### Non-radioactive phosphorylation assays

Custom N-terminally biotinylated peptides were purchased from Peptide Specialty Laboratories (PSL). For peptide substrate phosphorylation, recombinant CaMKII [100U/μl] was pre-activated (15 min at 30°C) in 1x kinase buffer in the presence of 2 mM Ca^2+^, 1.2 mM calmodulin and 1 mM ATP (all from New England Biolabs), and then mixed with peptide substrate (800 μM for ADP-Glo assay, Promega or 200 μM for MALDI-TOF). Following 1 h incubation at 30°C phosphorylation was assayed with ADP-Glo and MALDI-TOF-MS. Recombinant PKA-Cα (3U/μl, New England Biolabs) was incubated (30°C) with 200 μM peptide substrate, 1 μM ATP (PKLight, Lonza) or 1 mM (MALDI-TOF-MS) in 1x kinase buffer. Phosphorylation was assayed after 15 min (PKlight) or after 1 h (MALDI-TOF-MS). ADP-Glo and PKlight assays were performed according to manufacturer’s protocol with minor modifications. In brief, for ADP-Glo, 10 μl of phosphorylation reaction was mixed with 10μl of ADP-Glo reagent in white, flat-bottom, 96-well polypropylene plate. The plate was incubated at room temperature for 40 min, following application of 20 μl of Kinase Detection Reagent (KDR). Bioluminescence was measured after 40 min on a Centro LB 960 luminometer (Berdhold Technologies), with 0.5 s integration time. For PKLight, 20 μl of the phosphorylation reaction was mixed with 20 μl of ATP detection reagent in white, flat-bottom, 96-well polypropylene plate. The plate was incubated for 10 min, followed by bioluminescence measurement (0.1 s integration time). All reactions were performed at least in duplicate and each experiment was repeated at least 5 times. The data was normalized to „no-kinase” or „no-substrate” control levels and tested for significance with student’s t-test.

### MALDI-TOF mass spectrometry

Phosphorylated peptides (PSL) were desalted on the C18 ZipTips (Millipore). C18-bound peptides were eluted with 5 μl of the matrix (α-HCCA saturated in 33.3% acetonitrile/0.1%TFA), and applied on a MALDI target plate. Samples were dried at room temperature and analyzed on the Biflex III MALDI-TOF MS spectrometer (Bruker Daltonik GmbH), using the following parameters: IS1: 19 kV, IS2: 17.2 kV, lens: 8.8 kV, PIE: 200 ns, gating: maximum. Spectra were recorded in the linear positive mode at a laser frequency of 20 Hz, within a mass range from 1,000 to 6,000 Da. For each spectrum, at least 300 laser shots in 30 shot steps were collected. Each sample was analyzed at least in duplicate. Visual estimation of the mass spectra, raw data smoothing and baseline subtraction was performed using the FlexAnalysis 1.0 software. Obtained data was analyzed using the GraphPad Prism 5.0 software.

### DNA constructs and HEK-293 cell culture

HEK-293 cells were grown in DMEM medium supplemented with 10% FCS, 25 mM glucose, 6 mM L-glutamine, 0.1 mM non-essential amino acids, 1 mM sodium pyruvate (all from Invitrogen). CPEB3a-EGFP CMV expression plasmid was generated by inserting mouse CPEB3a ORF into EGFP-N1 vector backbone (Clontech). CPEB3a-FLAG and EGFP-FLAG were generated by inserting respectively CPEB3a and EGFP ORF into 3xFLAG-CMV-7.1 vector backbone (Sigma). mRuby-CaMKII was generated by replacing mGFP by mRuby (gift from J. Wiedenmann) in the mGFP-CaMKII plasmid (initially from EGFP-C1 vector (Clonetech)) [32]. All transfections were performed with Lipofectamine 2000 (Invitrogen) as directed by manufacturer.

### Immunocytochemistry in HEK-293 cells

HEK-293 cells were transfected with either the CPEB3a-EGFP vector only or both the CPEB3a-EGFP and mRuby-CaMKII vectors. 16 h post-transfection, the cells were transferred for 5 min in 1x Hank’s Balanced Salt Solution (HBSS, Invitrogen) only or stimulated in HBSS with 10 μM ionomycin and 5 mM CaCl_2_ [33]. Immediately thereafter, the cells were fixed in 4% PFA (0.1 M phosphate buffer, 4% sucrose, and 2 mM EGTA, pH 7.4, 37C) for 10 min. The cells were then washed twice in PBS, once in PBS with 0.1 M glycine, and permeabilized for 30 min in blocking solution (PBS, 0.1% Triton X-100, 2% normal goat serum). The mouse anti-αCaMKII (clone CBα2, 1:500; Invitrogen) antibody and the pCPEB3-S419/S420 antibody described here (1:100) were diluted in the blocking solution and incubated with the cells for 2 h. The cells were then rinsed three times in PBS and the following secondary antibodies were added for 45 min: Alexa Fluor 546 goat anti-mouse, Alexa Fluor 633 goat anti-rabbit. After 3x rinse in PBS, the coverslips were mounted on glass slides using Prolong Gold Antifade mounting reagent (Invitrogen). Images were acquired on LSM510-META Axioskop FS2 Plus (Carl Zeiss) confocal microscope, using a 63x 1.4NA oil immersion objective as described previously [33]. The mean intensity was measured in three chosen ROIs in 20 cells per condition over 8 different experiments. The ratio of phosphorylated CPEB3 average fluorescence over the CPEB3a-EGFP average signal for each cell was calculated, following normalization against the control values for each experiment. The statistical tests, detailed in the respective figure legends, were performed in MatLab.

### Immunocytochemistry on primary hippocampal neurons

Dissociated hippocampal neurons were prepared as previously described [32, 34]. Neurons at 11–15 DIV were transfected using Lipofectamine 2000 as described [32], with the CPEB3a-EGFP alone, or together with CaMKIIN-HA [35] plasmid. After 16 h, cells were pretreated for 1 h with either 10 μM KN93 (Calbiochem), 10 μM H89 (Tocris Bioscience), 300 nM OA (Sigma), 100 μM RP-cAMPS (Tocris Bioscience) or no drug. The coverslips were transferred in HBSS solution containing in 10 mM HEPES, 0.6 mM CaCl_2_ and 5 mM MgCl_2_ supplemented with the same drugs used for pre-incubation. After 5 min, cells were either transferred for 90 s in depolarizing solution consisting of HBSS supplemented with 40 mM KCl, 2.5 mM CaCl_2_, and 1 mM MgCl_2_ plus either 10 μM KN93, 10 μM H89, 100 μM RP-cAMPS or no drugs, or for 5 min in a standard solution consisting of HBSS supplemented with 2 mM glucose, 1.2 mM CaCl_2_ and 1 mM MgCl_2_ and containing the drugs used in pre-incubation plus 50 μM FS (Calbiochem) for the PKA activation experiments. Thereafter the cells were immediately fixed in 4% PFA. The immunofluorescence, image acquisition and analysis were performed as described for the HEK-293 cells.

### Kainate injections and quantitative RT-PCR

C57BL/6N mice (Charles River) at 3 months of age were injected intraperitoneally with kainite (2 mg/kg body weight) or sham injected with PBS. 30 min post injection, mice experiencing consistent *status epilepticus* were killed by cervical dislocation, decapitated and the brain was quickly removed, followed by dissection of the hippocampus. Total hippocampal RNA was extracted using RNAeasy Lipid Tissue Mini kit (Qiagen) with on-column gDNA digestion. Reverse transcription was done with SuperScript III RT (Invitrogen), using equal amount (1–2 μg) of RNA template, and random hexamers for priming. 0.5 μl of obtained cDNA was used for Taq-Man RT-PCR, carried out using Gene Expression Master Mix (Applied Biosystems), in Optical 384-Well Reaction Plate PCR plates (Applied Biosystems) with a 7900HT Fast Real-Time PCR System (Applied Biosystems), according to product recommendations. For each reaction, critical threshold cycle (Ct) value was determined using SDS 5.0 Software (Applied Biosystems). Efficiency of the primer/probes sets was calculated according to the serial dilution method [36, 37]. Data was analyzed with ∆∆Ct method. Oligonucleotide sequences: CPEB3 isoform multiplex PCR: 5’FAM/MGB—CAG GAG CTA TGG GCG GA-3’ (a/c isoform probe), 5’-VIC/MGB—CAC TTA ACA GTC GGT CTT-3’ (b/d isoform probe), 5’- ACC ATC CAG GAA CAG ATA ACA TTA TG-3’ (forward), 5’- CGT CTT CAA AGG GAA AGA GAG AAG-3’ (reverse), GAPDH: 5’- FAM–CAG TGC CAG CCT CGT CCC GTA GA—TAMRA-3’ (probe), 5’- GAG ACG GCC GCA TCT TCT TGT-3’ (forward), 5’-CAC ACC GAC CTT CAC CAT TTT-3’ (reverse). For ß-actin, a FAM/MGB Taq-Man assay was used (Applied Biosystems).

### Western blot

HEK-293 cells were transfected with CPEB3a-EGFP vector and 24 h post transfection medium was supplemented with 50 μM or 200 μM FS (New England Biolabs) or DMSO (as diluent control) for 1 h. After a brief rinse with ice-cold PBS, cells were harvested by scraping in a buffer containing 50 mM Tris-Cl, pH 7.5, 50 mM NaCl, 50 mM NaF, 2 mM EDTA, 2 mM NaVO_3_, 270 mM sucrose, 1% Triton X-100, 1% NP-40, supplemented with Complete Mini Protease Inhibitors and PhosStop Phosphatase Inhibitor Cocktail (both from Roche). Lysates were sonicated for 10 min and spun down (10 min / 10.000 g / 4°C) to pellet cell debris. Total protein content was assayed with a BCA (Pierce). Lysates were mixed with denaturing sample buffer and heated for 5 min at 95°C. Proteins (50 μg of total protein per loading) were separated by discontinuous SDS-PAGE in denaturing conditions [38] and electroblotted onto a PVDF membrane. Membranes were blocked in 5% skimmed milk powder in TBS buffer (pH 7.4) containing 0.05% Tween-20 (TBS-T) and incubated overnight at 4°C on an orbital shaker with primary antibodies: pCPEB3-S419/S420 (1:100), panCPEB3 (1:1000, ab10883, Abcam), α-tubulin (1:10000, T9026, Sigma). Membranes were washed with TBS-T and incubated for 1 hr with goat-anti-rabbit or goat-anti-mouse HRP-coupled secondary antibodies (1:10.000, both from Pierce). For pCPEB3-S419/S420 antibody, 5% BSA was used instead of milk powder for membrane blocking. West Dura HRP substrate (Pierce) was used to detect chemiluminescence, which was acquired with the Gene Gnome digital documentation system (Synoptics).

### Generation and validation of pCPEB3-S419/420 antibody

Rabbit polyclonal antibody was generated at Eurogentec GmbH. Bi-phosphorylated (pS419 and pS420) and monophosphorylated (pS419 or pS420) forms of the RRGRSSLFPFED peptide coupled to KLH were used for immunization. Obtained serum was purified positively against modified (phosphorylated) peptides, following depletion of the non-phosphospecific fraction against the unmodified peptide. **Assessing specificity:** biotynylated peptide substrate corresponding to CPEB3a/c was phosphorylated *in vitro* with recombinant PKA-C (New England Biolabs), immunoblotted, and detected with phospho-CPEB3a/c antibody following goat-anti-rabbit HRP-coupled secondary antibody (1:10.000, Pierce). Biotin-HRP was used to control for equal loading. **Assessing crossreactivity with CPEB2 and CPEB4:** recombinant vectors encoding CPEB2a-EGFP and CPEB3a-EGFP (EGFP-N1 vector backbone, Clontech) were transfected into HEK-293 cells. Cells were harvested by scraping in ice-cold kinase lysis buffer (1x kinase buffer (NEB), 1% Tx-100) supplemented with Complete Mini Protease Inhibitors and PhosStop Phosphatase Inhibitor Cocktail (Roche). Lysates were spun down (15.000 g / 10 min) to pellet the cell debris, incubated with PKA-C (3U/μl, NEB) and analyzed by Western blot.

### Immunoprecipitation and phosphorylation of FLAG-CPEB3a

HEK-293 cells expressing recombinant FLAG-CPEB3a protein were harvested 36 h post-transfection by scraping in ice-cold lysis buffer containing 10 mM HEPES pH 7.4, 200 mM NaCl, 2 mM EDTA, 1% Tx-100, Complete Mini protease inhibitors (1 tablet/10 ml) and Phos-Stop phosphatase inhibitors (1 tablet/10 ml). Lysates were sonicated for 10 min in ice-cold sonication bath and spun down (15 min/17.000 rcf). Supernatants were mixed with 50 μl of FLAG M2 agarose beads (Sigma), previously blocked with 3% BSA, and incubated for 2 h at 4°C on a rotator, followed by 5x washing with lysis buffer. CaMKII was preactivated at 30°C for 30 min in kinase reaction buffer (New England Biolabs) supplemented with 1.2 μM calmodulin and 2 mM CaCl_2_. Beads were mixed with kinase buffer containing 1 mM ATP and PKA-C_α_ (5000 U, New England Biolabs), pre-activated CaMKII (1000 U, New England Biolabs), or the corresponding buffer without enzyme. Sample was incubated for 30 min at 30°C with rotation and then mixed with 50 μl of NuPage reducing sample buffer and separated by SDS PAGE.

### Nanoflow electrospray LC-MS

Coomassie-stained proteins were excised and subjected to tryptic in gel digestion [39, 40]. In brief, proteins were reduced with 20 mM DTT, slices washed with 100 mM ammonium bicarbonate, and proteins alkylated with 40 mM iodoacetamide. The slices were washed again and dehydrated with acetonitrile. Gel pieces were dried in a vacuum concentrator and incubated with 400 ng sequencing grade trypsin at 37°C overnight. The peptide extract was dried in a vacuum concentrator and stored at -20°C. Dried peptides were dissolved in 8 μl 0.1% formic acid (solvent A). 2 μl were injected onto a C18 trap column (20 mm length, 100 μm inner diameter). Bound peptides were eluted onto a C18 analytical column (200 mm length, 75 μm inner diameter, both columns from NanoSeparations). Peptides were separated during a linear gradient from 1% to 45% solvent B (80% acetonitrile, 0.1% formic acid) within 18 min at a flow rate of 450 nl/min. The nano-HPLC was coupled online to an LTQ Orbitrap Velos mass spectrometer (Thermo Fisher Scientific). Peptide ions between 330 and 2000 m/z were scanned in the Orbitrap detector with a resolution of 30,000 (maximum fill time 400 ms, AGC target 10^6^). The eight most intense precursor ions (threshold intensity 5000) were subjected to collision induced dissociation with multiple stage activation (MSA, normalized collision energy 35, wide band activation) and fragment spectra recorded in the linear ion trap. After acquisition of eight MSA-spectra, the same eight precursor ions were subjected to higher energy collisional dissociation (HCD, normalized collision energy 42) and fragment spectra recorded in the Orbitrap detector. Fragmented peptide ions were excluded from repeat analysis for 20 s. Raw data processing and analysis of database searches were performed with Proteome Discoverer software 1.3 (Thermo Fisher Scientific). Peptide identification was done with an in house Mascot server version 2.3 (Matrix Science Ltd) and the Sequest search node in Proteome Discoverer. MS2 data were searched against mouse sequences from SwissProt (release 2012_07). Precursor ion m/z tolerance was 8 ppm, fragment ion tolerance 0.6 Da (CID) or 0.02Da (HCD). b- and y-ion series were included. Semitryptic peptides with up to two missed cleavages were searched. The following dynamic modifications were tested: phosphorylation of serine, threonine, and tyrosine, oxidation of methionine, propionamide or carbamidomethyl on cysteine, and acetylation of the protein N-terminus. Mascot and Sequest results from searches against SwissProt were sent to the percolator algorithm version [41] 1.17 as implemented in Proteome Discoverer. The PhosphoRS2.0 node was used for scoring of the phosphosite assignment [42]. Spectra with low scoring identifications and phospho-localizations were inspected manually.

## Results

### The alternatively spliced region of CPEB3 is a PKA and CaMKII target

**Fig 2:**
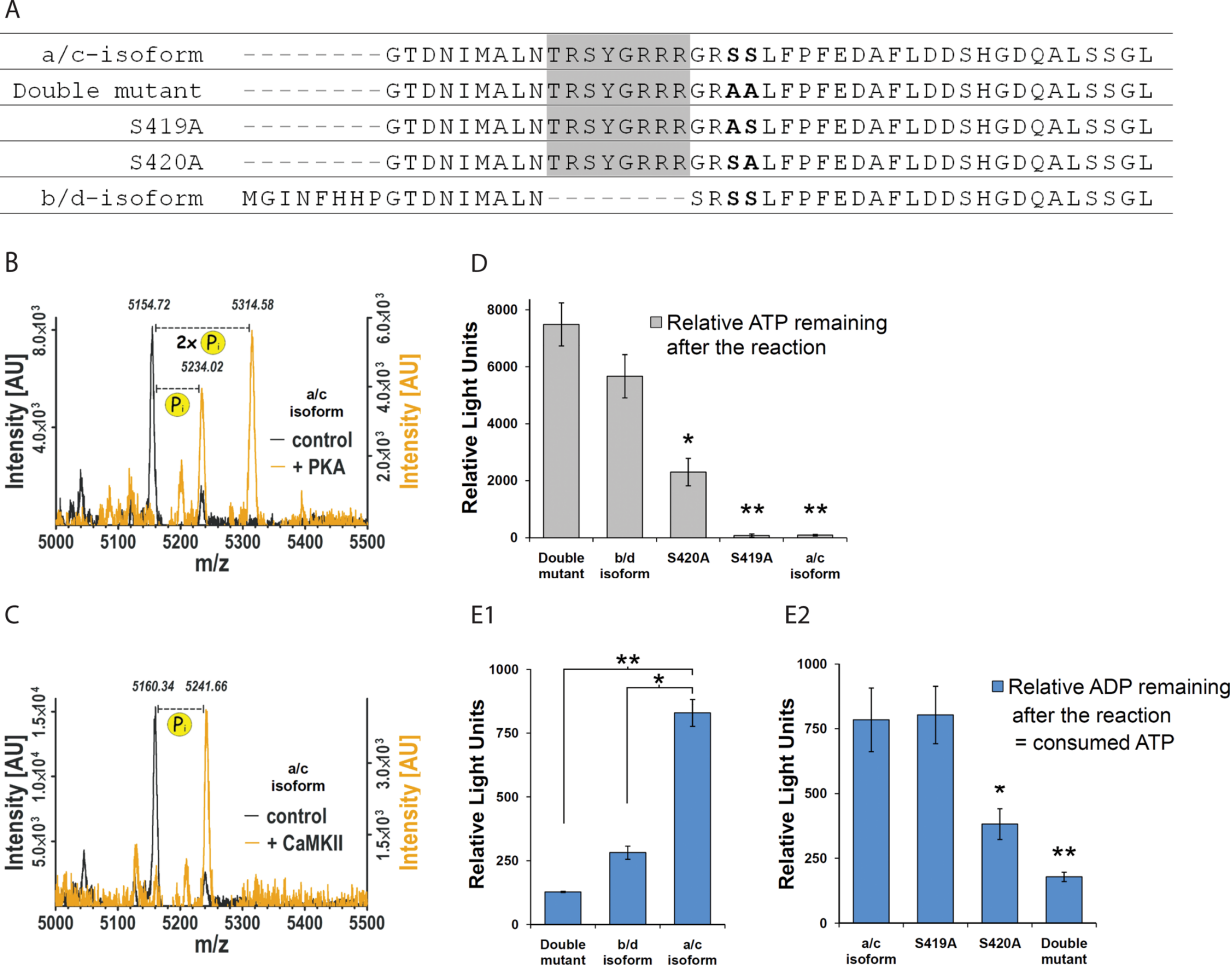
Non-radioactive phosphorylation assays. (**A**) Sequences of the synthetic peptides used for phosphorylation experiments in B, C, D, E. Alternatively spliced exon 7 (the B-region) was highlighted. Putative phosphorylation site is in **bold**. (**B**, **C**, **D**, **E**) *In vitro* phosphorylation of CPEB3 by PKA and CaMKII. (**B**) MALDI-TOF-MS analysis of CPEB3-derived peptides phosphorylated by PKA-C. A mass shift corresponding to incorporation of 1 or 2 phosphates is observed, as compared with no-kinase control. (**C**) MALDI-TOF-MS spectra of CPEB3-derived peptides phosphorylated by CaMKII. (**B, C**) orange trace–spectrum after phosphorylation; black trace–no-kinase control; m/z–mass to charge ratio; AU—arbitrary units. (**D**) Peptide phosphorylation by PKA-C (PKLight assay). The a/c-isoform peptide, S419A, and S420A mutant peptides are phosphorylated at a significantly higher rate compared to the double mutant, while the b/d-isoform phosphorylation was not significantly different. S420 is phosphorylated preferentially, as shown by decreased phosphorylation rate in case of S420A mutant peptide compared with S419A substitution (p<0.01). Values showing relative levels of unused ATP after phosphorylation reaction were normalized to no-substrate control levels (not shown). Error bars are SEM. *P<0.005, **P<0.001 (student’s t-test). (**E**) Peptide phosphorylation by CaMKII (ADP-Glo assay). (**E1**) The a/c-isoform peptide is phosphorylated at significantly higher rate compared with b/d-isoform and S419A/S420A double mutant. (**E2**) Serine 420 is phosphorylated preferentially, as shown by decreased phosphorylation rate in case of S420A mutant peptide. Values show relative ADP levels after phosphorylation reaction, normalized to no-substrate control levels (not shown). Error bars are SEM. *P<0.01, **P<0.001, ***P<0.05 (student’s t-test, n = 4).

To confirm our *in silico* predictions [21], we used a set of synthetic peptides containing serine residues S419 and S420 of CPEB3 (Fig 2A). The peptides included wild-type CPEB3a/c (with the B-region) and CPEB3b/d (without the B-region) peptides (respectively, containing and lacking a phosphorylation consensus motif), as well as the serine-to-alanine mutants (Fig 2A). For qualitative determination of phosphorylation, the a/c-isoform and the corresponding S419A/S420A double mutant peptides were phosphorylated *in vitro*, followed by Matrix-assisted laser desorption/ionization–time of flight (MALDI-TOF) mass spectrometry. PKA activity led to a shift of the a/c-peptide mass peak, as expected from addition of one (80 Da) or two (160 Da) phosphate (Pi) groups (Fig 2B, orange trace). No shift was observed for the S419A/S420A control peptide (Fig 2B, black trace). We then quantitatively compared the propensity of different peptide variants to phosphorylation, using non-radioactive, luciferase-based assays. The single alanine mutants (S419A and S420A) and the b/d-peptide were additionally included in the analysis. This allowed determining, respectively, which of the two serine residues are phosphorylated preferentially, and if the alternative splicing affects the CPEB3 protein propensity for phosphorylation. The a/c-peptide, the S419A, and the S420A mutant peptides were phosphorylated by PKA-Cα at a significantly higher rate compared to the S419A/S420A double mutant (Fig 2D), while there was no difference in b/d-peptide phosphorylation rate. PKA preferentially phosphorylated S420, as shown by the decreased phosphorylation rate in case of the S420A mutant peptide compared with the S419A substitution (p<0.01). No significant differences were observed between no-kinase and no-substrate control, to which the data were normalized (data not shown). In case of CaMKII, a MALDI-TOF mass shift was observed for a/c-peptide, and was equivalent to incorporation of one phosphate group (Fig 2C, orange trace). Quantitative analysis showed the a/c-peptide phosphorylated by CaMKII at a significantly higher rate than in the case of the S419A/S420A double mutant peptide and the b/d-peptide (p<0.001 and <0.01, respectively) (Fig 2E1). S420 was phosphorylated preferentially, as shown by the decreased phosphorylation rate in case of the S420A mutant peptide compared with the S419A substitution (p<0.05) (Fig 2E2). Interestingly, MALDI-TOF analysis of the peptide corresponding to the b/d-peptide showed a peak shift corresponding to incorporation of single (Pi) groups in case of PKA, but not in case of CaMKII (not shown). Considering the results of quantitative luciferase-based assays, we speculate that the a/c-peptide and the b/d-peptide are both phosphorylated by PKA, but at a significantly different rate.

### CPEB3a is phosphorylated by CaMKII and PKA in HEK-293 cells

**Fig 3:**
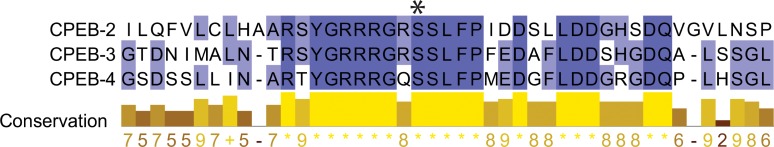
Multiple sequence alignment of the regions flanking S419/S420 of CPEBs-2-4. Phosphorylation site was marked with a black asterisk. Residue conservation score was calculated with Jalview software according to [61]. Color intensity and the score (1–11) in the “conservation index” (below the alignment) reflects the conservation of physicochemical properties of amino acids in the particular column of the alignment. *: conserved column (score 11, highest); +: all the amino acid physical properties conserved (score 10).

**Fig 4:**
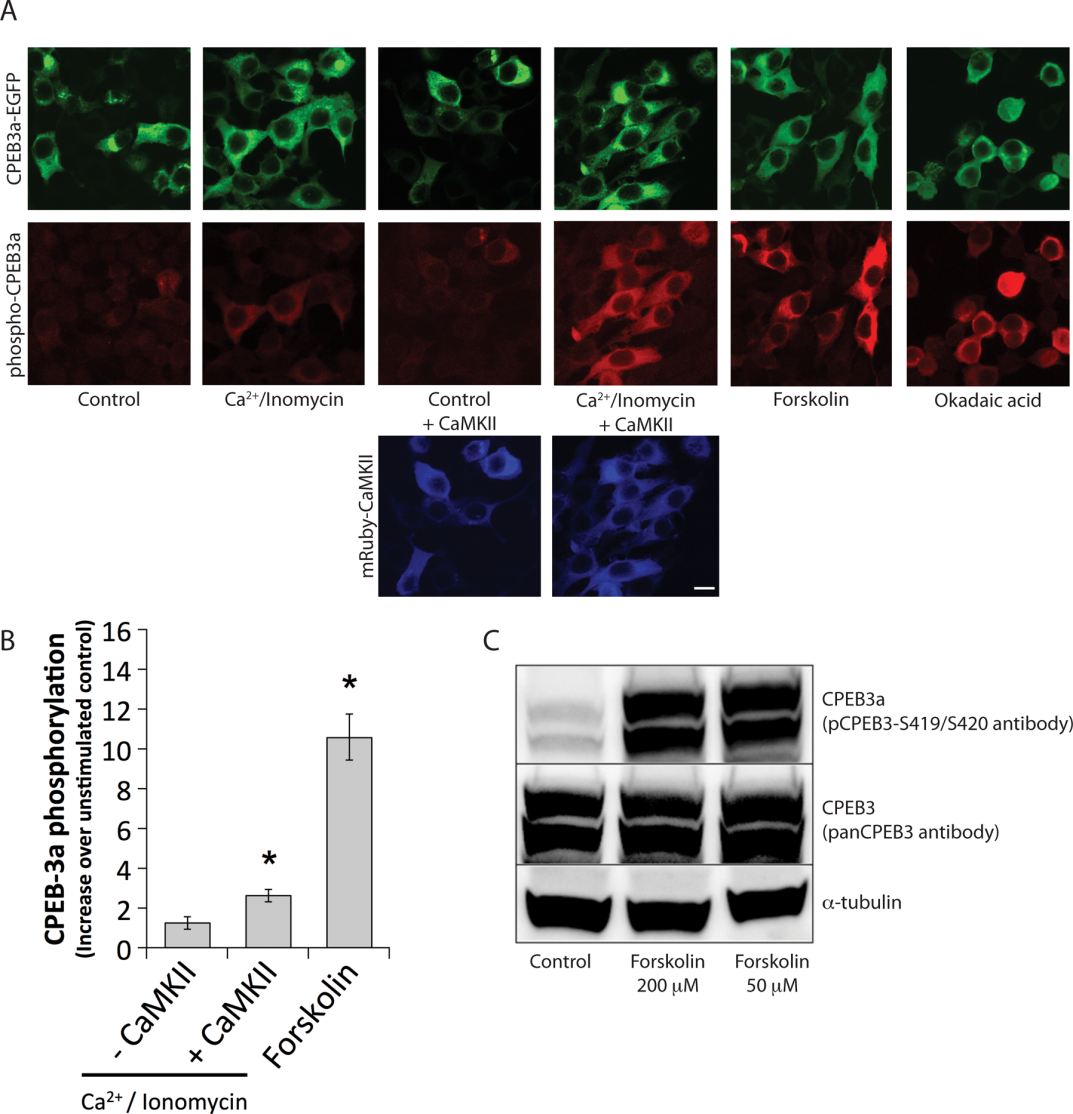
CPEB3a phosphorylation in HEK-293 cells. (**A**) Immunofluorescence of HEK-293 cells transfected with CPEB3a-EGFP or CPEB3a-EGFP and mRuby-CaMKII, and subjected to different stimulation paradigms. Forskolin (FS) stimulation led to a robust increase of phospho-CPEB3a signal. Ca2+/ionomycin stimulation led to a significant increase in phospho-CPEB3a only when mRuby-CaMKII was co-transfected. The scale bar represents 10 μm and applies to all photomicrographs. (**B**) Quantification of the immunofluorescence shown in A; n = 4–8 experiments with 20 cells per conditions, *p<0.05, Kruskal-Wallis followed by Tukey’s least-significant difference test. (**C**) A representative immunoblot showing increased phosphorylation state of CPEB3a protein in transiently transfected HEK-293 cells after FS stimulation (50μM and 200μM). PanCPEB3 and alpha-tubulin antibodies were used to control for equal loading.

To test the phosphorylation of CPEB3a in cells, we generated a phosphospecific antibody recognizing the phosphorylation consensus encompassing serines S419 and S420 (pCPEB3-S419/S420). The immunization strategy was designed so that the antibody would recognize the monophosphorylated forms of the protein (pS419 or pS420), as well as the bi-phosphorylated species (pS419 and pS420). The specificity of the antibody was confirmed on synthetic peptide substrate corresponding the CPEB3a/c isoform, and on the recombinant FLAG-tagged CPEB3a protein. Antibody pCPEB3-S419/S420 detected the synthetic peptide and the overexpressed FLAG-CPEB3a with high affinity and phosphospecificity (S1A and S1B Fig). We observed significant reactivity of the antibody with phospho-CPEB2a and no reactivity with phospho-CPEB-4a (S1C Fig). This was expected, as the similarity of the phosphorylation site is higher then in case of CPEB-4a (see also Fig 3). In HEK-293 cells transiently expressing CPEB3a-EGFP protein, co-transfecting with mRuby-CaMKII significantly enhanced phosphorylation following Ca^2+^/ionomycin stimulation compared with CaMKII-free controls (Fig 4A and 4B). Forskolin (FS) stimulation (50 μM for 1 h) also led to a large increase of CPEB3 phosphorylation, as compared with unstimulated controls (Fig 4A and 4B, p<0.05). The representative immunoblot showing CPEB3a phosphorylation increase after FS stimulation (50 μM and 200 μM for 1 h) is shown in Fig 4C. In the same conditions, we did not observe any immunoreactivity against CPEB-3a-KD (kinase-dead), and against CPEB-3b isoform (S1D Fig).

### CPEB3a is phosphorylated by CaMKII and PKA in hippocampal neurons

**Fig 5:**
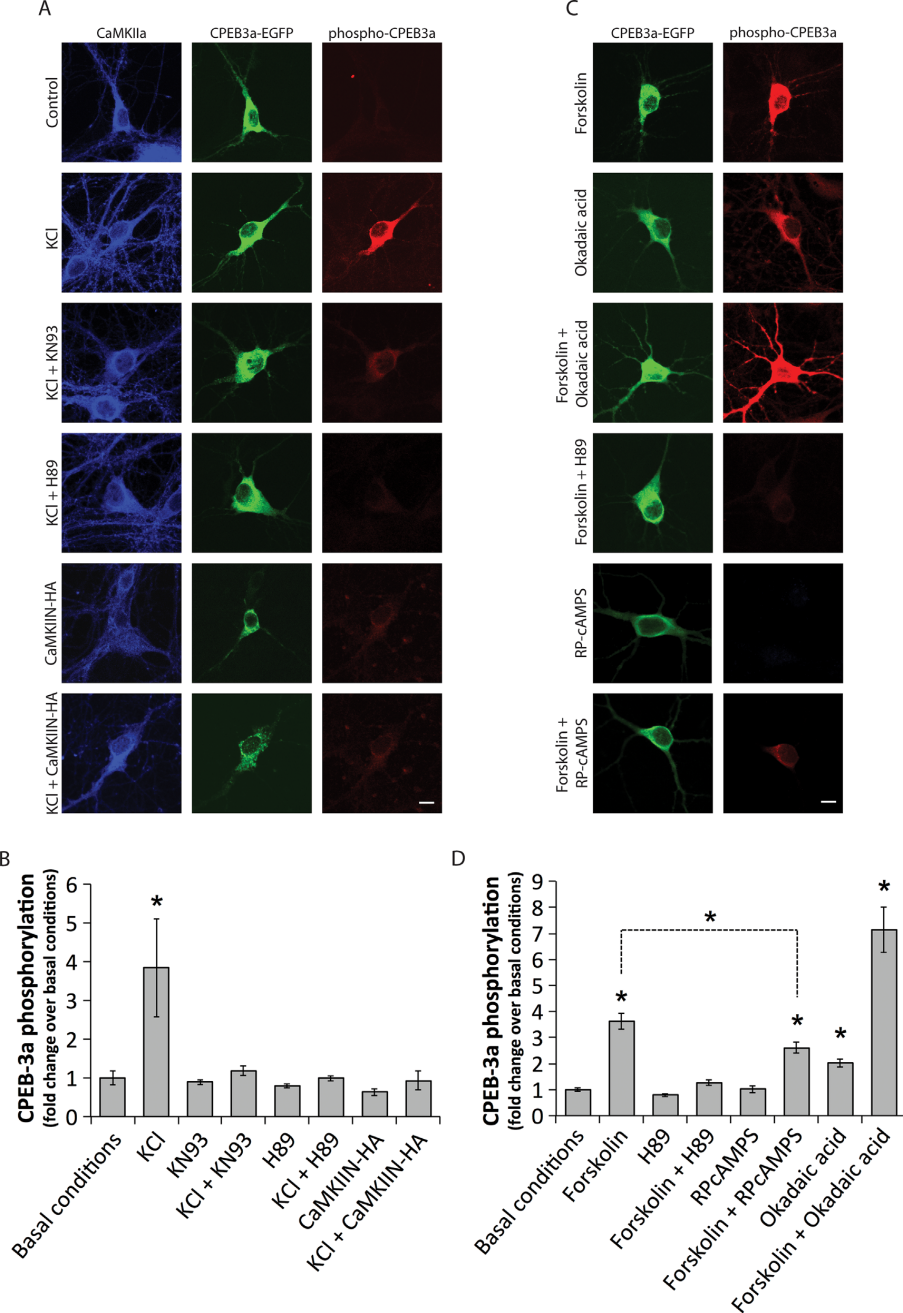
CPEB3a phosphorylation in cultured hippocampal neurons. Normalized ratio of phosphorylated CPEB3a (phospho-CPEB3a) fluorescence over total CPEB3a (CPEB3a-EGFP) fluorescence (n = 25–35, 3 experiments). (**A**) Immunofluorescence of neurons transfected with CPEB3a-mGFP subjected to a 40 mM KCl stimulation for 90 s, which leads to a marked increase in phospho-CPEB3a signal. One hour pretreatment with 10 μM KN93 or 10μM H89 in the stimulation medium reverts this increase. Co-transfection of the natural CaMKII inhibitor (CaMKIIN-HA) prevents the increase in CPEB3a phosphorylation. (**B**) Quantification of the ratio of phospho-CPEB3a over CPEB3a-mGFP signals in A, which shows a significant increase of CPEB3a phosphorylation following KCl stimulation only, which is not seen in neurons treated with KN93, H89, or those co-transfected with CaMKIIN-HA; n = 10–35, at least 3 experiments per condition. (**C**) Immunofluorescence of neurons transfected with CPEB3a-mGFP and subjected to either 5 min 50 μM FS stimulation or 1 h 300 nm OA stimulation, which both cause a robust increase in phospho-CPEB3a signal. Pretreatment with 10 μM H89 or 100 μM RP-cAMPS for 1 h partially reverts the effect of FS. (**A**, **C**) The scale bar represents 10 μm and applies to all photomicrographs. (**D**) Quantification of the ratio of phospho-CPEB3a over CPEB3a-mGFP signals in C, which shows a significant increase of CPEB3a phosphorylation following FS, OA and combined FS + OA treatments. Increase in phosphorylation induced by FS is partially reversed by cAMPS-Rp treatment; n = 22–59, 2–5 experiments per condition. (**A**, **B**, **C**, **D**) *p<0.05, Kruskal-Wallis followed by Tukey’s least-significant difference test.

Primary hippocampal neurons expressing CPEB3a-mGFP and depolarized with 40 mM KCl for 90 s showed significantly higher phosphorylation of CPEB3a compared with unstimulated controls (Fig 5A and 5B, p<0.05). Pre-incubating with CaMKII and PKA inhibitor (respectively, 10 μM KN93 and 10 μM H89, 1 h each, or co-transfection of a competitive CaMKII inhibitor CaMKIIN-HA [35]) prevented KCl-induced phosphorylation (Fig 5C and 5D, p<0.05). FS (50 μM, 5 min)-induced phosphorylation of CPEB3a was prevented by pre-treatment with H89 (10 μM) or RP-cAMPS (100 μM). Treatment with okadaic acid (OA) (0.3 μM), a PP1/PP2A/PP2B inhibitor [43], led to a significant increase in phosphorylated CPEB3, which was further increased when combined with FS treatment (5 min) (Fig 5C and 5D, p<0.05).

### Kainate stimulation alters the ratio of CPEB3 isoform expression

**Fig 6:**
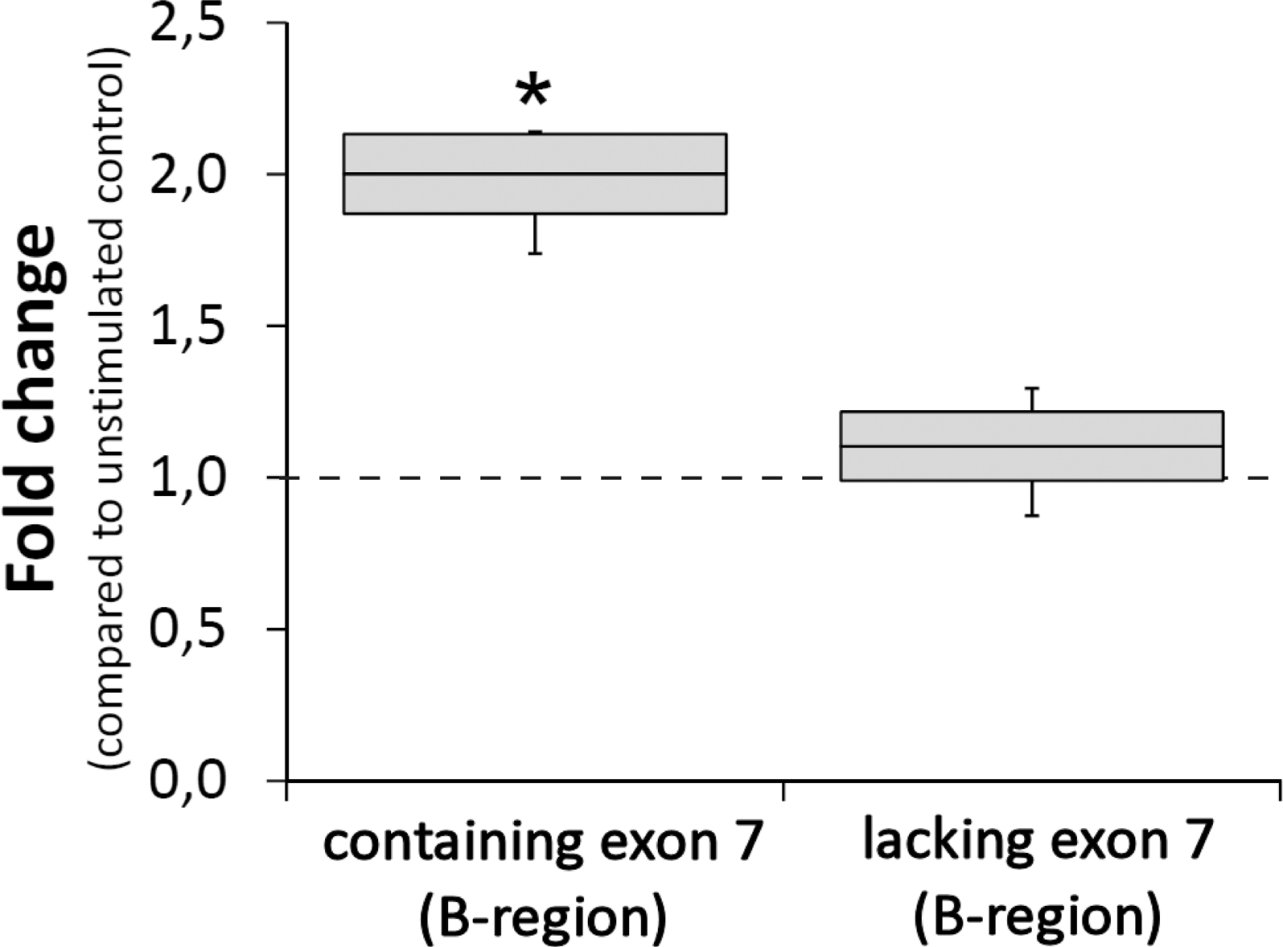
qRT-PCR analysis of CPEB3 isoforms of hippocampal mRNA after intraperitoneal kainate injection. The mRNA levels of isoforms with the B-region increased 30 min after kainate-induced *status epilepticus*, while the levels of the isoforms lacking the B-region remained unchanged. n = 4 animals = 8 hippocampi (per condition). *p<0.05, student’s t test.

We previously showed by *in-situ* hybridization (ISH) that hyperactivity leads to upregulation of CPEB3 in principal neurons of the hippocampus and cortex [21]. Since neuronal stimulation leads to increased phosphorylation of CPEB3 at S419/S420, we wanted to determine if it alters the alternative splicing of exon 7. To this end, we designed a TaqMan qRT-PCR assay, for simultaneous quantification of B-region-containing and B-region-lacking isoforms of CPEB3. Inducing seizures in mice by intraperitoneal injection of kainate, a potent agonist of non-NMDA (N-methyl-D-aspartate)-type glutamate receptors), led to upregulation (to 201% ± 12% of control levels, n = 4) of specifically the splice variants containing the phosphorylation consensus site (the B-region). The levels of mRNA corresponding to splice variants lacking the B-region remained unchanged (Fig 6).

### CPEB3a is phosphorylated at multiple positions

To look for other phosphorylation sites on CPEB3a, a FLAG-tagged CPEB3a was transiently expressed in HEK-293 cells and then immunoprecipitated (S2 Fig). Part of the immunoprecipitate was phosphorylated with recombinant PKA and CaMKII, following analysis by LC-MS. Seventeen different phosphopeptide species were identified, covering 11 potential phosphorylation sites in two regions (residue numbering refers to full length 716aa CPEB3a protein, Uniprot Q7TN99-1): S285 to S299 and S419 to S446. The latter overlaps with the alternatively spliced middle region of the CPEB3 protein, where phosphorylation by PKA, PKB, CaMKII and RPS6K kinases was previously predicted [20, 21]. A total of 121 spectra were confidently assigned to phosphopeptides. Manual validation of spectra could not resolve all ambiguous localization cases but groups of potential phosphorylation sites could be identified. The extent of phosphorylation was estimated by counting peptide spectrum matches (PSMs) of each ambiguity group. PSMs from three PKA phosphorylation experiments and from experiments without additional phosphorylation (endogenous phosphorylation control) were averaged. Phosphorylation of control sample was detected on S291 and on S443 or S444. pS443 and pS444 could not be distinguished due to their proximity. Phosphorylation on S291 was also unambiguously identified in PKA and CaMKII samples. S419 or S420 were phosphorylated after additional CaMKII treatment and to a lesser extent by PKA treatment. The proximity of these sites hampered unambiguous localization of phosphorylation. S432 and S444 might be modified *in vitro* by CaMKII, but the number of PSMs associated with this site was low. Localization of phosphorylation to S439, S443, and S444 was complicated by the ambiguity of spectra produced by mono- and bisphosphorylated peptides containing these amino acids. Many longer monophosporylated peptides containing S419 to T446 were identified after PKA treatment. Exact localization of the phosphosites was not possible but fragment ion spectra indicated a probable modification on (S419 or S420) or (S443, S444) rather than S432, S439, S440, or T446. Evidence for double phosphorylation on (S419 or S420) and on (S443 or S444) was found after CaMKII treatment. In PKA treated samples three spectra of triply phosphorylated peptide species appeared indicating phosphorylation on S419 and/or S420 along with S444 and possibly S432. Triply phosphorylated species were not found in other samples. Results are summarized in Table 1.

**Table 1:** Summary of mass spectrometric evidence for phosphorylation of CPEB3a by PKA, CaMKII and endogenous kinases present in HEK-293 cells.

Position	Peptide motif	PKA	CamKII	No kinase (endogenous phosphorylation)
285	VGVGVPsPLNPIS			
291	SPLNPIsPLKKPF	++	++	+
298	PLKKPFsSNVIAP			
299	LKKPFSsNVIAPP			
291 or 298 or 299		(+)	+	(+)
419	GRRRGRsSLFPFE			
420	RRRGRSsLFPFED			
419 or 420		+	++	
419 and 420			(+)	
432	DAFLDDsHGDQAL		(+)	
439	HGDQALsSGLSSP			
440	GDQALSsGLSSPT			
443	QALSSGLsSPTRCQN			
444	ALSSGLSsPTRCQNG			
443 or 444				(+)
(419 or 420) and (443 or 444)			+	
419 or 420 or 439			(+)	
419 or 420 or 443 or 444		++		
446	SGLSSPtRCQNGE			

Each line contains the position and surrounding sequence of an amino acid residue found to be phosphorylated in LC-MS. If the phosphosite could not be assigned unambiguously for a spectrum, all possible positions are given.

(+): 1–2 peptide spectrum matches (PSMs) +: 3–5 PSMs ++: > 5 PSMs

### The B-region of CPEB3 is a putative target of multiple kinases

To complement our phosphorylation analysis of the CPEB3 alternatively spliced region, we screened a synthetic a/c-isoform peptide against the panel of 190 S/T kinases (S1 Text). This revealed a robust *in vitro* phosphorylation of this region, which was not observed for the corresponding peptide lacking the B-region (data not shown). The analysis revealed CPEB3a/c to be a likely substrate for such kinases as the tumor suppressor WNKII, Ribosomal Protein S6 Kinase (RPS6K) and Calcium/Calmodulin-dependent Protein Kinase IV (CaMKIV) (S3 Fig). The complete results for all kinases used in the screening are shown in S1 Table.

## Discussion

Phosphorylation was previously shown to regulate CPEB activity. *Xenopus* CPEB is phosphorylated by Aurora kinase, leading to increased polyadenylation of maternal mRNA [44, 45]. In brain, mouse CPEB-1 (a homolog of *Xenopus* CPEB) is phosphorylated by Aurora and upon NMDA receptor activation, via Aurora and CaMKII kinase [7, 8, 46]. Consistent with an established role in neuronal plasticity [47–49], overexpression of kinase-dead CPEB-1 in Purkinje neurons (T171A and S177A mutant) impairs cerebellar plasticity and motor functions in mice [50]. In contrast to CPEB-1, much less information is available on how the activity of the remaining CPEBs is regulated.

The CPEB3 protein in mouse brain occurs in four splice variants: a, b, c, and d, differing by the presence (or absence) of the B- and C-regions (Fig 1). The B-region (corresponding to exon 7 of the CPEB3 gene) harbours the kinase recognition site. To date, phosphorylation of this region has only been predicted *in silico* [20, 21, 25]. Here we present evidence that PKA and CaMKII phosphorylate CPEB3 isoforms containing the B-region, but not the isoforms lacking it. Similarly to RNA binding domains, this region is highly conserved between CPEBs-2-4 (Fig 3). Moreover, the splicing pattern of CPEB3 is similar to that of CPEB2 and CPEB4 [20, 25], consistent with the fact that the proteins are paralogs. We have previously shown CPEB2 splice isoforms being differentially expressed in cell types of the mouse hippocampus [25]. Interestingly, individual single CA1 neurons often expressed different CPEB2 isoforms [25]. How is this splicing modulated and what is the significance of this phenomenon?

As CPEB3 is implicated in synaptic plasticity [16, 27, 51], we tested if the neuronal stimulation *in vivo* leads to changes in ratios of CPEB3 isoforms. To this end we have designed a quantitative multiplex RT-PCR assay, to discriminate between splice variants containing- and lacking the B-region. We then performed systemic injections of kainate (an agonist of non-NMDA type glutamate receptors) in mice to induce epileptic seizures. Such treatment led to a shift in mRNA levels of CPEB3 splice variants containing the B-region, but not the ones lacking it, consistent with our previous observations [21]. Certain conditions, such as increase of neuronal activity, might therefore lead to increased inclusion of the B-region. As this region harbors a phosphorylation consensus, it would render the CPEB3 pool capable of being activated by phosphorylation.

To explore this further, and to facilitate future studies, we have raised an antibody detecting phosphorylation of CPEB3a at serine residues S419/S420. With this antibody we showed for the first time in cells, that CPEB3 splice variants containing the B-region are targets of PKA and CaMKII. We tested CPEB3a phosphorylation in HEK-293 cells and in cultured hippocampal neurons. In each experimental paradigm, FS (adenylyl cyclase activator) led to a robust increase in phospho-CPEB3 levels, which was reversed by the PKA inhibitor H89. As FS also indirectly activates CaMKII by triggering intracellular calcium release, we cannot exclude its contribution to CPEB3 phosphorylation in neurons [52], which would confirm our *in silico* predictions [21], and would be in line with the recently reported role of CPEB3 in LTP and memory [51, 53]. CaMKII increased CPEB3a phosphorylation on S419/S420 after Ca^2+^/ionomycin stimulation in HEK-293 cells. KCl depolarization of primary neurons transfected with CPEB3a showed CaMKII- and PKA-dependent S419/S420 phosphorylation. Addition of OA led to increased phospho-CPEB3a, suggesting a rapid dephosphorylation under basal conditions. We speculate that depolarization might lead to activation of CaMKIIα and PKA, but also to activation of phosphatases (e.g. PP1), as suggested by the additive effect of co-application of FS and OA. In a parallel study we showed that interfering with CPEBs-1-4 function by overexpressing an N-terminally truncated CPEB-1 (lacking the N-terminal phosphorylation domain) leads to deficits in LTP and spatial reference memory (Martin Theis, unpublished observations). Considering the overlap mRNA target specificity between CPEBs, such a transgene likely interferes with the function of CPEBs2-4. Indeed, a similar memory impairment phenotype was observed in CPEB3 KO mice, due to upregulation of AMPA receptors in postsynaptic density (PSD) [51]. In addition, as both CaMKII and PKA influence synaptic plasticity and are downstream targets of AMPA receptor signaling, it is likely that these kinases modulate CPEB3 function similarly as they do for CPEB-1 [48]. CPEB3 KO mice show increased activation of CaMKIIα [53]. CaMKIIα is required for synaptic tagging in late-phase LTP (L-LTP) [54], and was shown to cause hyper-phosphorylation of CPEB-4 (CPEB3 paralog) *in vivo* in purified post PSD fractions [27]. Of note, *Aplysia* CPEB (ApCPEB) is involved in long-term facilitation (LTF) by forming a prion-like multimers upon neuronal (serotonin) stimulation [55, 56]. ApCPEB is a homolog of mammalian CPEB3, which also contains a glutamine-rich prion-like domain. Such an aggregation could be an interesting mechanism for translation activation in dendrites, possibly also involving modulation by protein kinases. Indeed, CPEB3 was shown to have prion properties [56, 57] and to mediate local protein synthesis required for LTP maintenance in the hippocampus [58]. To date, the role of phosphorylation in these processes has not been addressed.

In addition to the four isoforms we studied (a, b, c, d), additional CPEB3 isoforms have been found in mouse retina [31]. We found the CPEB2 splicing pattern to be cell type specific [25]. As the number of known biological functions of CPEBs is growing (cell senescence and cancer [10, 59], synaptic plasticity [51], transcriptional regulation [30, 60]), the potential regulatory mechanisms involving CPEB3 may be biologically highly relevant. Given the cell-type specificity of CPEB3 isoform expression, it is conceivable that alternative splicing is one of such mechanisms. The sequence similarity between CPEBs-2-4 is >98% in the RBD. The splicing pattern of CPEB2 and CPEB-4 is similar to that of CPEB3: they all contain the C-region and the B-region, and in each case the latter overlaps with the phosphorylation consensus site [20, 25]. The B-domain is proximal to the RNA binding domain, and is highly conserved between CPEBs-2-4 (Fig 3). Therefore, CPEBs-2-4 probably share the propensity for phosphorylation by the same array of protein kinases (this would also explain observed crossreactivity of our pCPEB3-S419/S420 antibody with the CPEB2a protein). With this in mind, to complement our study, we performed detailed *in vitro* analysis of CPEB3 phosphorylation. By LC/MS we confirmed that S419 and S420 are the primary targets of phosphorylation, at least by PKA and CaMKII. In addition, the analysis revealed two other sites undergoing phosphorylation in HEK-293 cells, which might have a regulatory role as well. Altogether, this detailed analysis of CPEB3 phosphorylation *in vitro* and in cells expands our understanding of the CPEB family, while the pCPEB3-S419/S420 antibody may be a useful tool to study the regulation of CPEB3 in physiological and pathological conditions.

## Supporting Information

S1 Fig Immunoblot validation of the pCPEB3-S419/S420 polyclonal antibody directed to S419/S420. (PDF)

S2 Fig Immunoprecipitation of FLAG-CPEB3a protein expressed in HEK-293 cells. (PDF)

S3 Fig In vitro validation of CPEB3a-derived peptide phosphorylation by CaMKIV, Ribosomal Protein S6 Kinase (RPS6K) and With-No-Lysine kinase II (WNK2). (PDF)

S1 Table Results of 190 S/T kinases screening against a peptide derived from CPEB3a isoform containing the B-region. (PDF)

S1 Text Supplementary methods. (PDF)

## Abbreviations

ApCPEB: Aplysia Cytoplasmic Polyadenylation Element Binding protein
CaMKIIα: Calcium/Calmodulin-dependent Protein Kinase II alpha isoform
CPE: Cytoplasmic Polyadenylation Element
CPEB: Cytoplasmic Polyadenylation Element Binding protein
FS: Forskolin
LC-MS: liquid chromatography–mass spectrometry
MALDI-TOF: Matrix-assisted laser desorption/ionization–time of flight
OA: Okadaic acid
p38MAPK: p38 Mitogen-Activated Protein Kinase
RPS6K: Ribosomal Protein S6 Kinase
ISH: In-situ hybridization
