# Supplementary material for: New Phosphospecific Antibody Reveals Isoform-Specific Phosphorylation of CPEB3 Protein
Source: PLoS One. 2016 Feb 25;11(2):e0150000. doi: 10.1371/journal.pone.0150000 (PMC4767366; doi:10.1371/journal.pone.0150000)
Supplement: S1 Fig — (PDF) [file pone.0150000.s001.pdf]

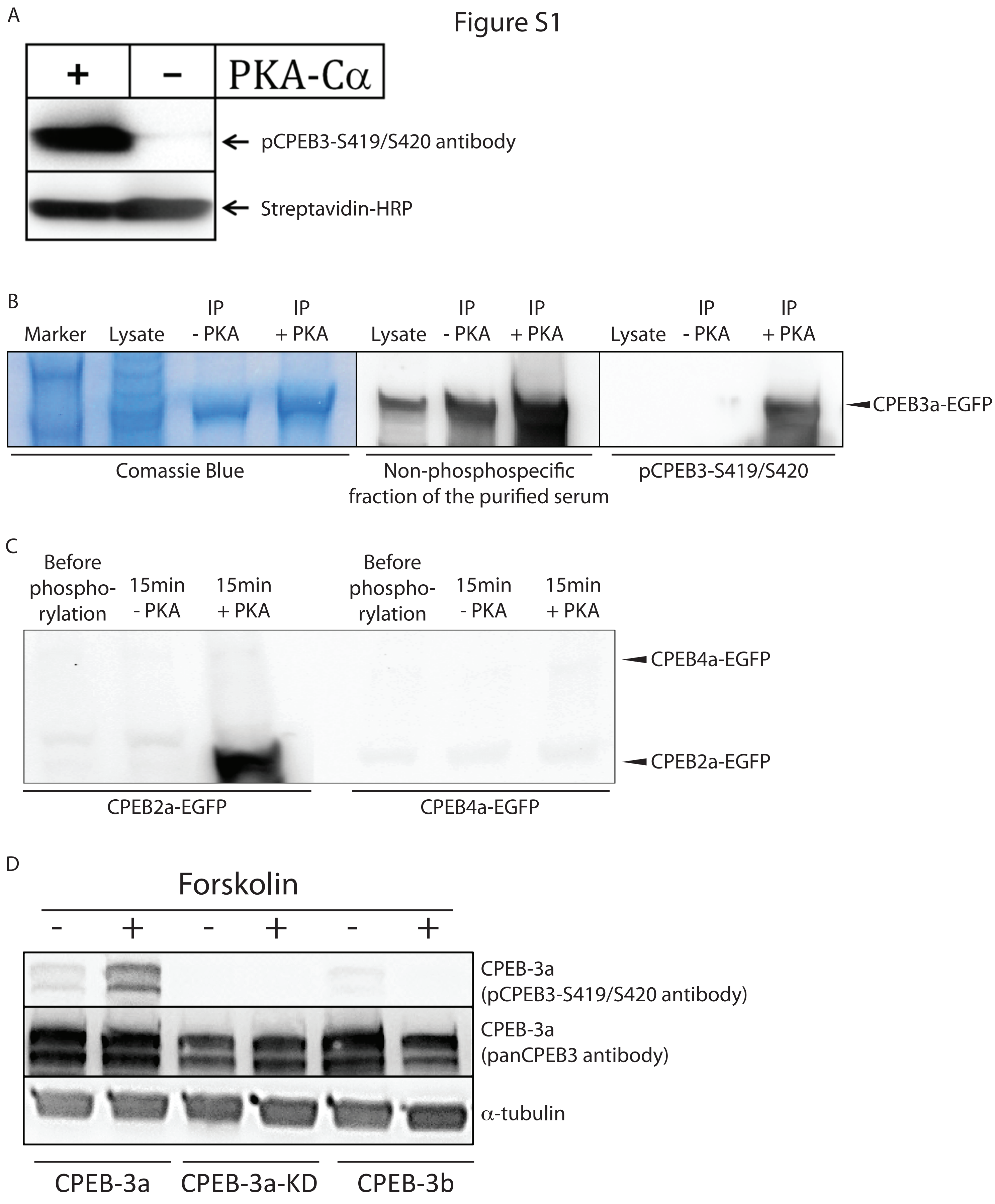

Figure S1: Immunoblot validation of the pCPEB3-S419/S420 polyclonal antibody directed to S419/S420 of CPEB3a.

(A) pCPEB3-S419/S420 antibody recognized specifically the phosphorylated form of the CPEB3 peptide corresponding the a/c isoforms. Streptavidin-HRP, detecting biotinylated peptide was used to control for equal loading.

(B) pCPEB3-S419/S420 antibody detected only the phosphorylated form of a full-length recombinant CPEB3a protein.

(C) pCPEB3-S419/S420 antibody cross-reacted with CPEB2a (left) but not with CPEB4a (right). Control sample was treated identically, except that the kinase was omitted.

(D) Immunoblot showing no pCPEB3-S419/S420 antibody activity upon Forskolin stimulation against kinase dead (KD) CPEB-3a mutant and CPEB-3b isoform.
