# Supplementary material for: New Phosphospecific Antibody Reveals Isoform-Specific Phosphorylation of CPEB3 Protein
Source: PLoS One. 2016 Feb 25;11(2):e0150000. doi: 10.1371/journal.pone.0150000 (PMC4767366; doi:10.1371/journal.pone.0150000)
Supplement: S2 Fig — (PDF) [file pone.0150000.s002.pdf]

Figure S2

	Coomassie							
sample	IP						input	
	CPEB-3a-FLAG PKA		CPEB-3a-FLAG CaMKII		CPEB-3a-FLAG no kinase	EGFP-FLAG	CPEB-3a-FLAG	EGFP-FLAG
lane	1	M	2	M	3	4	5	6

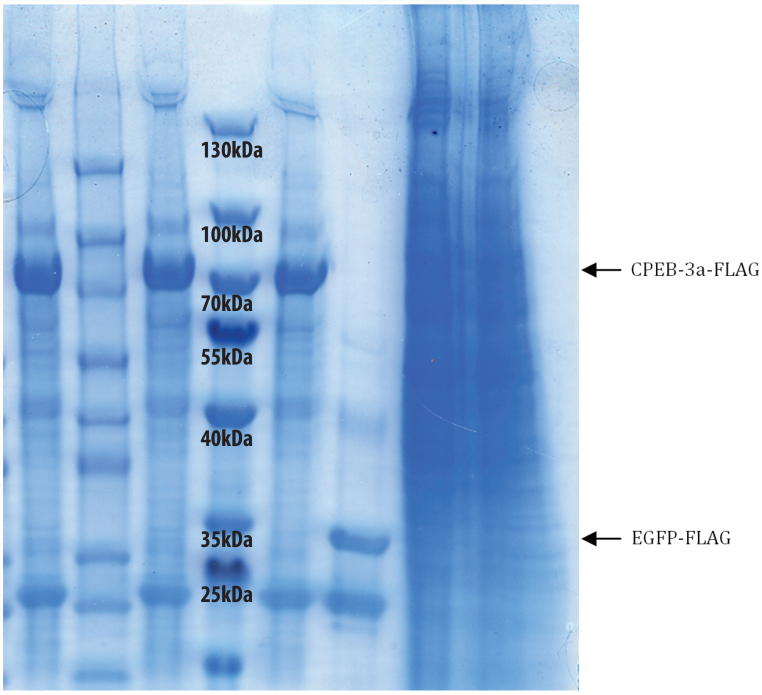

	Western Blot					
sample	IP				input	
	CPEB-3a-FLAG PKA	CPEB-3a-FLAG CaMKII	CPEB-3a-FLAG no kinase	EGFP-FLAG	CPEB-3a-FLAG	EGFP-FLAG
lane	1	2	3	4	5	6

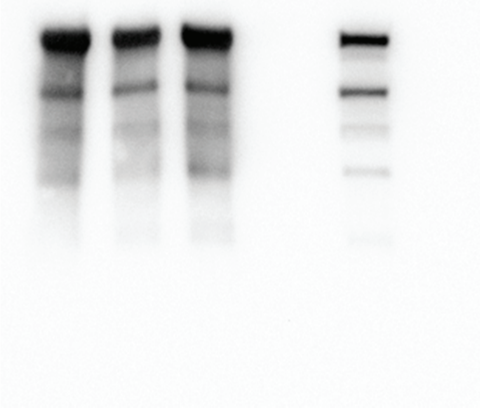

Figure S2: Immunoprecipitation of FLAG-CPEB3a protein expressed in HEK-293 cells. Right – Coomassie staining, left – corresponding immunoblot. FLAG-EGFP fusion protein was used as a control.
