# Supplementary material for: New Phosphospecific Antibody Reveals Isoform-Specific Phosphorylation of CPEB3 Protein
Source: PLoS One. 2016 Feb 25;11(2):e0150000. doi: 10.1371/journal.pone.0150000 (PMC4767366; doi:10.1371/journal.pone.0150000)
Supplement: S3 Fig — (PDF) [file pone.0150000.s003.pdf]

Figure S3

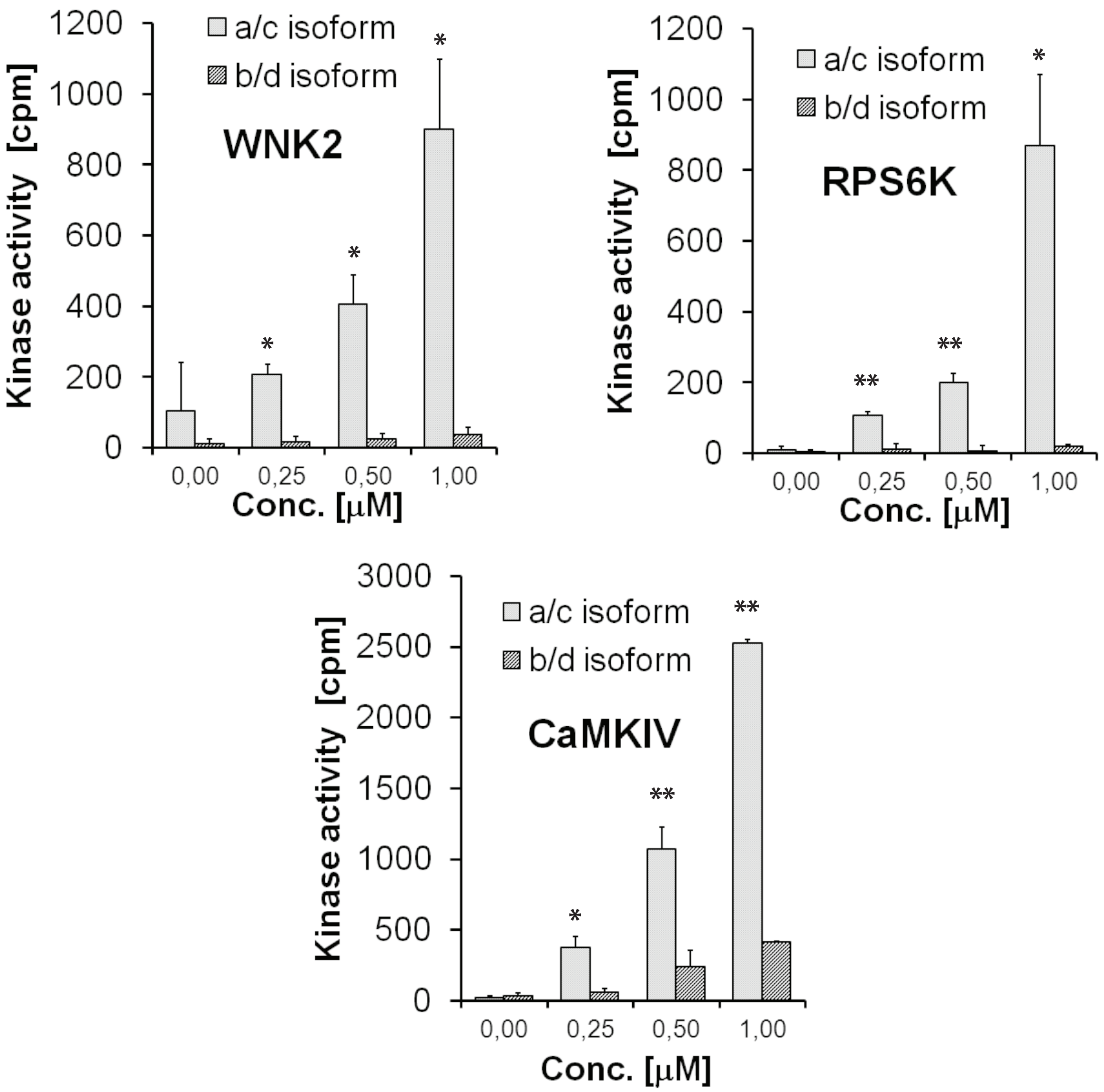

Figure S3: In vitro validation of CPEB3a-derived peptide phosphorylation by CaMKIV, Ribosomal Protein S6 Kinase (RPS6K) and With-No-Lysine kinase II (WNK2).
Error bars are SEM. *P<0.05, **P<0.01 (student's t-test).
