# Supplementary material for: New Phosphospecific Antibody Reveals Isoform-Specific Phosphorylation of CPEB3 Protein
Source: PLoS One. 2016 Feb 25;11(2):e0150000. doi: 10.1371/journal.pone.0150000 (PMC4767366; doi:10.1371/journal.pone.0150000)
Supplement: S1 Table — (PDF) [file pone.0150000.s004.pdf]

Table S1

			A	B	C
#	Kinase	A-B-C	Activity	Kinase Background	Substrate Background
1	CAMK4	5536	5593	51	6
2	PAK2	2813	2837	18	6
3	PAK3	2795	2838	37	6
4	SGK2	2543	2551	2	6
5	PKA	2334	2351	11	6
6	PAK1	1981	2024	37	6
7	WNK2	1562	1568	0	6
8	CAMK1D	901	945	38	6
9	NEK2	896	973	71	6
10	IRAK4	867	879	6	6
11	MST2	744	750	0	6
12	ARK5	708	877	163	6
13	RPS6KA3	675	718	37	6
14	TSSK1	659	689	24	6
15	IKK-beta	627	670	37	6
16	DAPK1	622	637	9	6
17	CHK1	601	714	107	6
18	PASK	598	684	80	6
19	DAPK2 (+CaM)	514	566	46	6
20	NEK1	472	478	0	6
21	PKC-delta	434	477	37	6
22	MST1	429	435	0	6
23	DAPK3	417	516	93	6
24	IKK-epsilon	403	440	31	6
25	CAMK2D	378	394	10	6
26	PKC-eta	372	381	3	6
27	WNK3	355	361	0	6
28	PKC-zeta	350	427	71	6
29	SNARK	344	448	98	6
30	RPS6KA1	333	339	0	6
31	RPS6KA2	331	342	5	6
32	HRI	330	355	19	6
33	SAK	315	439	118	6
34	PRKG1	308	357	43	6
35	SGK3	302	310	2	6
36	CAMKK2	289	308	13	6
37	NEK7	276	310	28	6
38	MARK1	274	345	65	6
39	DYRK3	274	280	0	6
40	ROCK2	259	286	21	6
41	MST4	246	323	71	6
42	TSF1	230	256	20	6
43	DYRK1A	222	240	12	6
44	PHKG1	218	261	37	6
45	RPS6KA5	200	255	49	6
46	Aurora-A	190	231	35	6
47	MAPKAPK3	185	191	0	6
48	COT (Lot018)	180	251	65	6
49	IRAK1	179	231	46	6
50	PAK4	172	196	18	6
51	PKC-beta2	171	177	0	6
52	ACV-RL1	168	181	7	6
53	RPS6KA4	166	224	52	6
54	MAP4K4	166	187	15	6
55	CDC42BPB	148	161	7	6
56	EIF2AK2	144	150	0	6
57	RIPK2	140	146	0	6
58	AKT2	134	202	62	6
59	CK1-alpha1	130	231	95	6
60	PLK1	129	175	40	6
61	PRKX	128	152	18	6
62	RPS6KA6	128	149	15	6
63	ACV-R1B	124	150	20	6
64	EIF2AK3	123	203	74	6

			A	B	C
#	Kinase	A/B	Activity	Kinase Background	Substrate Background
6	TAOK2	122	168	40	6
6	PKC-nu	122	155	27	6
6	PKC-alpha	118	124	0	6
6	CAMK2B	106	135	23	6
6	ZAK	105	116	5	6
7	PAK7	105	111	0	6
7	TSK2	102	108	0	6
7	STK23	101	190	83	6
7	CDK2/CycA	101	115	8	6
7	PKC-epsilon	100	134	28	6
7	CLK2	98	104	0	6
7	AKT3	96	102	0	6
7	TBK1	94	135	35	6
7	NEK3	92	98	0	6
7	STK17A	92	98	0	6
8	SNK	88	123	29	6
8	mTOR	82	128	40	6
8	MINK1	82	125	37	6
8	CK1-gamma2	82	95	7	6
8	PKC-gamma	81	87	0	6
8	PBK	80	86	0	6
8	AMPK-alpha1	79	105	20	6
8	Aurora-B	78	134	50	6
8	PIM3	77	98	15	6
8	PRK2	77	83	0	6
9	MARK3	74	204	124	6
9	SNF1LK2	74	98	18	6
9	PKC-theta	74	80	0	6
9	DMPK	72	103	25	6
9	MELK	72	90	12	6
9	IKK-alpha	69	134	59	6
9	PHKG2	69	121	46	6
9	MAPKAPK5	67	73	0	6
9	MST3	66	109	37	6
9	MYLK2	66	84	12	6
1	CLK3	63	69	0	6
1	AKT1	62	79	11	6
1	GRK2	60	128	62	6
1	PLK3	60	97	31	6
1	GSK3-beta	59	77	12	6
1	PDK1	57	63	0	6
1	CLK1	56	197	135	6
1	MAP4K2	55	61	0	6
1	PKC-iota	54	75	15	6
1	CDK1/CycE	51	67	10	6
1	CDK1CycB1	47	73	20	6
1	ACV-R1	46	52	0	6
1	PKC-beta1	46	52	0	6
1	PKC-mu	46	52	0	6
1	TAOK3	44	70	20	6
1	LRRK G2019S	43	49	0	6
1	CDK5/p35NCK	41	61	14	6
1	CK1-delta	40	66	20	6
1	CK1-gamma3	39	45	0	6
1	PRKD2	39	45	0	6
1	SGK1	37	66	23	6
1	CAMK2A	37	45	2	6
1	NEK4	36	42	0	6
1	RON	36	42	0	6
1	PIM2	35	41	0	6
1	CDK1/CycA	33	49	10	6
1	GRK6	33	42	3	6
1	NEK6	31	65	28	6
1	NEK11	30	36	0	6

		A	B	C	
#	Kinase	A/B	Activity	Kinase Background	Substrate Background
129	B-RAF wt	28	39	5	6
130	S6K-beta	27	54	21	6
131	CDK5/p25NCK	26	67	35	6
132	CDC42BPA	25	45	14	6
133	CDK9/CycT	25	36	5	6
134	NEK9	23	103	74	6
135	PAK6	22	52	24	6
136	S6K	22	36	8	6
137	GRK4	21	49	22	6
138	SRPK1	20	33	7	6
139	CHK2	19	48	23	6
140	ERK1	19	40	15	6
141	RAF1 DYDY	19	31	6	6
142	NIK	18	148	124	6
143	CK1-gamma1	17	55	32	6
144	CK2-alpha1	16	36	14	6
145	GRK5	15	30	9	6
146	GRK7	12	18	0	6
147	VRK1	11	55	38	6
148	SRPK2	11	18	1	6
149	ROCK1	11	17	0	6
150	Aurora-C	8	52	38	6
151	CK2-alpha2	8	18	4	6
152	ASK1	7	42	29	6
153	TGFB-R1	7	21	8	6
154	CDK6/CycD1	5	18	7	6
155	CDK7CycH	4	24	14	6
156	CDK4/CycD3	0	6	0	6
157	ERK2	0	6	0	6
158	CDK3/CycE	-5	12	11	6
159	PRK1	-6	28	28	6
160	JNK3	-6	12	12	6
161	JNK2	-7	21	22	6
162	DYRK1B	-7	-1	0	6
163	MKK6SDTD	-7	-1	0	6
164	p38-alpha	-7	-1	0	6
165	p38-beta	-7	-1	0	6
166	PCTAIRE1	-8	69	71	6
167	CDK2/CycE	-8	9	11	6
168	B-RAF VE	-11	18	23	6
169	CDK8/CycC	-12	-6	0	6
170	GRK3	-13	15	22	6
171	LIMK1	-14	39	47	6
172	MAP4K5	-18	59	71	6
173	NLK	-19	-1	12	6
174	p38-delta	-21	-6	9	6
175	GSK3-alpha	-23	33	50	6
176	CDK4/CycD1	-23	0	17	6
177	TGFB-R2	-27	10	31	6
178	CK1-epsilon	-29	-3	20	6
179	PIM1	-30	7	31	6
180	MEK1 wt	-31	6	31	6
181	BRSK1	-32	-3	23	6
182	STK33	-33	44	71	6
183	PRKG2	-33	10	37	6
184	TTK	-34	126	154	6
185	MARK2	-34	18	46	6
186	DCAMKL2	-35	2	31	6
187	HIPK3	-40	15	49	6
188	JNK1	-41	-1	34	6
189	p38-gamma	-83	-6	71	6
190	HIPK1	-93	37	124	6

S1 Table. Results of 190 S/T kinases screening against a peptide derived from CPEB3a isoform containing the B-region.
