# Supplementary material for: New Phosphospecific Antibody Reveals Isoform-Specific Phosphorylation of CPEB3 Protein
Source: PLoS One. 2016 Feb 25;11(2):e0150000. doi: 10.1371/journal.pone.0150000 (PMC4767366; doi:10.1371/journal.pone.0150000)
Supplement: S1 Text — (PDF) [file pone.0150000.s005.pdf]

Kaczmarczyk, Labrie-Dion et al.

Isoform-specific phosphorylation of CPEB3 - supplementary methods

Supplementary methods

Peptide synthesis

Peptide synthesis (Peptide Specialty Laboratories) was performed with a Biotin-PEG NovaTag resin (Merck Chemicals) on a continuous flow synthesizer. After trifluoric acid (TFA) cleavage the peptides were purified by HPLC and verified by MALDI-TOF MS. Peptides were dissolved in sterile water.

In vitro serine/threonine (S/T) kinase screen

The S/T kinase phosphorylation profile of full-length recombinant CPEB3a (synthesized as a custom service at Eurogentec) and CPEB3-derived peptides was performed at ProQinase (Freiburg). Initial screening against a panel of a panel of 190 serine/threonine kinases was performed at 1 µM substrate concentration, using a Streptavidin FlashPlate-based radiometric detection method. 10 µl of kinase solution and 40 µl of buffer/ATP/peptide mixture were pipetted on a 96 well, V-shaped, polypropylene assay plate. The reaction cocktails contained 60 mM Na-HEPES, pH 7.5, 3 mM $MgCl_2$, 3 mM $MnCl_2$, 3 µM $NaVO_3$, 1.2 mM DTT, 1 µM ATP/[γ-33P]-ATP (8.3 x 10^5 cpm per well), protein kinase (1-400 ng/50 µl) and peptide (5 µg/50 µl). Where applicable, mixes additionally contained 1 mM $CaCl_2$, 4 mM EDTA, 5 µg/ml Phosphatidylserine and 1 µg/ml 1.2-dioleyl-glycerol, or 1 µg/ml calmodulin and 0.5 mM $CaCl_2$, or 1 µM cGMP. One well of each assay plate was used for a buffer/substrate control containing no enzyme. The assay plates were incubated at 30°C for 60 min. Subsequently, the reaction cocktails were stopped with 20 µl of 4.7M NaCl/35 mM EDTA. The reaction cocktails were transferred into 96-well StreptaFlashPlatesTM (PerkinElmer), followed by 30 min incubation at room temperature on a shaker. Subsequently the plates were aspirated and washed three times with 250 µl of 0.9% NaCl. Incorporation of 33Pi was determined with a microplate scintillation counter (Microbeta, Perkin Elmer). For the raw data analysis, previously determined kinase background values were normalized to the counts used in the experiment and subtracted from the corresponding raw data obtained with each kinase. Additionally, the background value of the peptide sample (median of two measurements) was subtracted from each raw value. Positive hits were verified in a similar way, at three peptide concentrations (1 µM, 0.5 µM and 0.25 µM) in triplicate. The differences in phosphorylation propensity between the peptides were tested for significance by student's t-test.
